# The use of music for children and adolescents living with rare diseases in the healthcare setting: a scoping review study protocol

**DOI:** 10.12688/hrbopenres.13280.1

**Published:** 2021-05-14

**Authors:** Simona Karpaviciute, Alison Sweeney, Aimee O‘Neill, Sandra McNulty, Thilo Kroll, Suja Somanadhan

**Affiliations:** 1 Children’s Health Ireland at Temple Street Children’s University Hospital, Temple St, Dublin 1, D01 XD99, Ireland; 2 School of Psychology, Trinity College Dublin, the University of Dublin College Green, Dublin 2, D02 PN40, Ireland; 3 UCD Centre for Interdisciplinary Research, Education and Innovation in Health Systems, UCD School of Nursing, Midwifery and Health Systems, University College Dublin, UCD Health Sciences Centre, Belfield, Dublin-4, B113A, Ireland

**Keywords:** Music, music-based intervention, music therapy, music for health, rare disease, children and adolescents, health and well-being, healthcare

## Abstract

**Background: **Interest in the application of music in the health, social care and community contexts is growing worldwide. There is an emerging body of literature about the positive effects of music on the well-being and social relationships of children and adult populations. Music has also been found to promote social interaction, communication skills, and social-emotional behaviours of children with medically complex care needs. Despite significant advancements in the area, to the authors’ knowledge, this is the first scoping review to investigate the evidence for using music therapy and music-based interventions for children living with rare diseases in the healthcare setting. Therefore, the purpose of this study is to conduct a scoping review of the literature to map out the existing studies about the use of music therapy and music-based interventions with children who have rare diseases in the healthcare setting. This review will also identify gaps in current knowledge and use of these interventions.

**Method: **This study follows the Joanna Briggs Institute’s methodology for scoping reviews, utilising Arksey and O’Malley’s six-stage scoping review framework: 1) identifying the research question; 2) identifying relevant studies; 3) study selection; 4) charting the data; 5) collating, summarising and reporting results; and 6) consulting with relevant stakeholders step. A comprehensive search will be conducted in CINAHL Complete; MEDLINE Complete; Psychology and Behavioral Sciences Collection; and PubMed Central databases. A search strategy with selected inclusion and exclusion criteria will be used to reveal a wide range of evidence. This study will include quantitative, qualitative and mixed research methods studies published in English from 2010 to 2020.

## Introduction

Rare diseases affect a small number of people compared to the general population. In Europe, a rare disease is defined as a disease or disorder affecting fewer than five in 10,000 of the European population ( European Commission, n.d). Between 27 and 36 million people reside in Europe with one of the 8000 distinct diseases, which means that rare diseases create a significant public health and social care issue in Europe. Although rare diseases are individually rare, collectively, they are common in society. 50–75% of all rare diseases affect children, and the challenges faced by children and their families are often similar regardless of the specific diagnosis ( Eurordis, 2009).

Children with rare diseases have multiple ongoing complex health and developmental issues due to the complex, chronic nature of rare diseases, which often cause intellectual and physical disability that place substantial demands requiring multidisciplinary input and regular hospitalisations ( Anderson *et al*., 2013; Elliott & Zurynski, 2015; HSE, 2019; Kuo *et al.*, 2011; vand der Lee *et al.*, 2007). Rare disorders significantly impact children’s overall health and well-being, and development, not only physically but also psychosocially ( Eurordis, 2009; HSE, 2019).

Evidence suggests that arts-based activities are effective in reducing adverse physiological and psychological outcomes. Recent findings from a review published by the World Health Organisation ( Fancourt & Finn, 2019) demonstrated that the arts for health activities were used in disease prevention and health promotion (for instance, support child development, encourage health-promoting behaviours, help to prevent ill health and support caregiving) and management and treatment (e.g. support care for people with chronic conditions and many more). The “Arts on Prescription” has been used for around two decades in the United Kingdom as part of broader social prescribing schemes showing the benefits for mental health, chronic pain, management of complex and long-term conditions, social support and well-being (Drinkwater, Wildman, Moffatt, 2019 cited in Fancourt & Finn, 2019). Engagement with creative arts-based activities has been used as a vehicle to improve capacity for self-reflection and understanding of oneself and others ( Stuckey & Nobel, 2010).

There is substantial evidence demonstrating the positive impact of music on an individual’s health and well-being ( Hagemann *et al*., 2019; Impellizzeri *et al*., 2020; Irons *et al*., 2020; Ørjasęter *et al*., 2018; Picard *et al*., 2014; Tamplin *et al*., 2014).

There is growing interest in music therapy and music-based interventions (MT&MBI) in healthcare settings, both in adult and child populations. The use of MT&MBI has been associated with substantial psychosocial benefits. For example, exposure to music has been found to reduce stress and anxiety in pregnancy and build mother-infant relationships ( Corbijn van Willenswaard *et al*., 2017; Wulff *et al*., 2015), and it has helped manage anxiety and stress ( Franzoi *et al*., 2016; Kesselman *et al*., 2016) and pain associated with diagnostic procedures and treatments ( Calcaterra *et al*., 2014; Sabzevari *et al*., 2017; van der Heijden *et al*., 2015).

In children and young people, especially those living with disabilities such as autism or those who experience developmental delays, music has been shown to improve speech and communication, social interaction and emotional skills ( Groß *et al*., 2010; Janzen & Thaut, 2018), motor function ( Bharathi *et al*., 2019) and social outcomes ( Ghasemtabar *et al*., 2015; LaGasse, 2017; Spiro & Himberg, 2016; Wan *et al*., 2010b). Other research has found benefits of music in terms of supporting well-being and coping abilities for children and young people with mental health conditions ( Grebosz-Haring & Thun-Hohenstein, 2018; Sollia, 2015), epilepsy ( Lin *et al*., 2014), cancer ( Ahmadi, 2013), neurological disorders ( Wan *et al*., 2010a) and anorexia nervosa ( Bibb *et al*., 2015), as well as building a supportive environment in the palliative care context ( Archie *et al*., 2013; Porter *et al*., 2017).

Although considerable evidence indicates that music contributes to the child’s physical and mental well-being, the evidence on the use of MT&MBI for children living with rare diseases is minimal. Similarly, very little is known about how MT&MBI are used to explore the children’s and young people experiences of living with an illness.

For this reason, our cross-disciplinary team of health researchers (including arts for health researcher) decided to conduct this scoping literature review study focused primarily on the use and the impact of MT&MBI on the health and well-being of children living with a rare disease in the healthcare settings. To develop music-based interventions for this specific target group, we need to investigate the existing findings, underline the research gaps, and draft the recommendations for the researchers and policymakers to inform future research and practice.

## The aim of the scoping review

The purpose of this paper is to conduct a scoping review of the literature to map out the existing studies about the use of MT&MBI with children who have rare diseases in the healthcare setting. The study also aims to explore and summarize the diversity of these studies, answering the following questions:

- What were the main characteristics of MT&MBI?
- In what healthcare settings have these studies been carried out?
- Who were the MT&MBI targeted at?
- What types of research designs methods were used?
- What theoretical frameworks were used to guide intervention development and evaluation?
- What was the nature and type of interventions?
- Which outcomes have been described in the literature?
- What were the roles and significant impacts of MT&MBI on participants’ health and well-being?

## Methods

The knowledge regarding the general benefits of music on the individuals and the community’s health and well-being are well-established, yet data about its impacts on children living with rare diseases remain limited. There is very little evidence on the use of MT&MBI to support health and well-being and explore lived experiences of children living with rare diseases. Thus, we have decided to conduct a scoping review, which is the optimal methodology to reflect on our study aim. It will follow Arksey & O’Malley’s (2005) original five-stage scoping review framework (identify the research question; identify relevant studies; select the studies; chart the data; collate, summarize and report the results) and more recent refinements to the method proposed by Levac *et al*. (2010) and the Joanna Briggs Institute (JBI) methodology for scoping reviews ( Peters *et al*., 2020). This study will involve the following six stages in undertaking a scoping review: 1) identifying the research question; 2) identifying relevant studies; 3) selecting studies; 4) charting the data; 5) collating, summarising and reporting the results and 6) consulting with relevant stakeholders ( Levac *et al*., 2010). This scoping review will emphasise the current evidence base. It will significantly contribute to the rare diseases research and policy focused on arts and music as a research method, effective arts-based participatory tool for research involving children, and tool to support individual-and community-level well-being.

This study protocol will outline how we will address each of the six stages.

***I. Identifying the research question***

In this study, we carefully planned the research question and the focus of the scoping review. To identify the research question, we searched and designed our key search terms to capture the most appropriate body of literature.

In identifying the study question, the study team identified key concepts that encompass the cross-disciplinary study's scope. Five team members were involved in this process (including an arts for health researcher, music therapist and experienced researcher investigating the quality of life of children living with a rare disease and the care relationships between the children and their carers). The key concepts were as follows: music therapy and music interventions, health and well-being, children and adolescents living with rare diseases/conditions, healthcare setting. The study's key focus will be MT&MBI as a research method and/or tool to support children’s health and well-being within a healthcare setting and explore their lived experiences. We will analyse the existing literature in the field to answer if and how diverse forms of MT&MBI are being used in healthcare settings for children living with rare diseases.

The study question of our research is: “What are the roles and benefits of MT&MBI as a tool for researching children living with a rare disease in healthcare settings?”

***II: Identifying relevant studies***

In the beginning, three researchers (including a music therapist) identified the databases for the literature search process. A comprehensive search will be conducted in the following databases: CINAHL Complete; MEDLINE Complete; Psychology and Behavioral Sciences Collection; and PubMed/PMC. These sources include esteemed journals in the area of health and social care. Additionally, if the number of articles suitable for the inclusion in the study is very small, searches will be conducted in the APA PsychINFO; ERIC; Lenus Irish Health Research Repository; and Google Advanced Search/Google Scholar databases too. The inclusion criteria will be based on the population–concept–context ( **PCC**) framework recommended by the JBI ( Peters *et al.*, 2020). Discussion among all research team members regarding exclusion and inclusion criteria at the start of the scoping review process occurred, and two researchers made the final decision. Table 1 offers an overview of the selection of the articles (eligibility) criteria for this study.

**Table 1.** Overview of articles selection criteria.

Aspects of articles selection criteria	Articles inclusion criteria	Articles exclusion criteria
**Population** **Target group** **characteristics**	Human; Children living with rare diseases; Studies working with participants aged between four and 16 years.	Any other population; Any other group of participants; Any other age group.
**Concept** **Type of activity**	Discussion or application of music therapy and music-based interventions, technique/session	Other art forms
**Context** **Study location**	Healthcare setting (at any location both locally and internationally)	Other settings
**Type of articles**	Studies from peer-reviewed journals	1) Non-peer reviewed documents and grey literature (such as book chapters, book reviews, policy reports, bibliographies, technical reports, conference proceedings and reports, accessible dissertations and theses, research protocols and reports, commentaries, editorials and letters); 2) Duplications.
**Accessibility**	Open access and full-text publications	Restricted access publications (paid)
**Year of publication**	Articles published between January 2010 and October 2020	Any publication outside these years
**Language**	Publications are written in English	Any other language
**Research methods**	Papers using qualitative, quantitative, mixed research methods and case study examples; Articles using the music therapy, music-based interventions as a research method, research tool, or initiative.	Articles with very poor quality of the study design and findings presentation; Articles that focus on other arts forms.

***III. Search strategy and study selection***

Following Arksey & O’Malley’s (2005) framework, we will outline the vocabulary we will apply within our search strategy to find the most valuable literature relevant to our study question. In designing our search strategy, our team (with interdisciplinary and cross-disciplinary experience) will discuss the areas of teamwork, agree on discussions (in the main steps of the article writing process) and joint decisions, as noted by Levac *et al*. (2010).

To select the final keywords, we will carry out an initial literature search in a relevant database to see what definitions are dominant (searching article titles, abstracts, keywords relating to our study aims). At the end, a comprehensive set of keywords will be identified and used in our study. During the search phase, if any additional significant keywords are identified, we will include these in our searches across all databases. At the end, our search strategy will be carefully developed by our research team and if needed, in consultation with the librarian. Experts will approve carefully selected keywords. At least three researchers (including arts for health researcher, music and art therapists) will be involved in the search strategy design and listing the keywords relevant to music-focused research. The literature search strategy will be carried out using several search terms; keywords are overviewed in Table 2.

**Table 2.** The literature search strategy: keywords.

Type of arts activity / art form	Study population	Health condition	Study setting
music OR music therapy OR music activity OR music session OR music intervention OR music for health OR music and health	child OR children OR youth OR youngster OR kid OR teen OR teenager OR adolescent OR preteen OR junior OR immature OR young people OR young	chronic disease OR chronic condition OR chronic illness OR rare disease OR rare illness OR rare condition OR complex needs	healthcare OR health care OR hospital OR hospice OR clinical OR outpatient OR inpatient OR outpatient care OR inpatient care OR rehabilitation OR recovery OR healing OR treatment

At least three independent reviewers will screen the listed studies by title and abstract. They will subsequently conduct a full-text review based on the pre-specified inclusion/exclusion criteria relevant to our research question. The reviewers will meet at different stages of the review process to discuss/report any challenges, clarify uncertainties, make necessary refinements to the search strategy following the recommendations provided by Levac *et al*. (2010), and the studies selection step. All disagreements will be solved, finding a shared agreement. Should any major disagreement arise regarding the inclusion or exclusion of studies that cannot be resolved by consensus, an additional reviewer will be invited to make the final decision.

***IV. Charting the data: initial characteristics***

A chart will be used for data extraction influenced by the guidelines from the JBI Reviewer’s Manual ( Peters *et al*., 2020). This consists of particular study aspects and related questions to inform the charting process. It will support the next stage of the review process: collating, summarising, and reporting the results, identifying themes. The process of charting data will be open to integrate new characteristics of the selected studies if these will enhance the data analysis and help to reflect on our study questions. Table 3 shows a primary table of charting characteristics and associated aspects of data extraction.

**Table 3.** Initial table of the overview of study findings: the characteristics of the studies.

Studies characteristics	Focus on the following aspects
**Authors, Year**	Who are the authors of the study? What year was the study published?
**Journal**	What is the title / name of the journal?
**Country**	Where was the study published? Where was the study conducted?
**Target group**	Who were the participants / who was the target audience of the study: identifying group/-s and how many persons participated in the study? What were the characteristics of the participants (socio-demographic, health related, etc.)?
**Aim**	What was / were the aim /-s of the study?
**Setting**	Where was the study conducted (identifying the setting / context, for e.g. identifying the hospital department; was it a clinical, outpatient, rehabilitation, other setting)?
**Activity**	What were the forms of music applied in this study? What was the duration of the programme (how many sessions, how often)? Who was the music activity lead (art therapist, musician, artist, clinician, researcher, other professional)? What was the content of the music activity? What form /-s of music was / were used in the study? Was the music combined with other art forms? Was the music element used at one or several parts of the study? Did the aspects of music activity change in the study?
**Participant recruitment**	How were the participants invited to take part in the study? Was it voluntary participation or not? Were there any collaborations with NGOs, patient advocacy institutions, universities, other sectors?
**Research methods**	What types of research methods were applied in the study: qualitative, quantitative, mixed, arts-based research methods? Was a music professional (or other specialists) part of the research team / involved in developing the study / music aspect of the study and how?
**Ethical approval**	Was there any evidence of ethical approval? Were there any specific ethical considerations / issues?
**Data analysis**	How was the data analysed: what methods were used? What were the parameters for data analysis?
**Main results**	What were the main results of the study? Was there any focus / what was the impact of music on participants’ health and well-being? What was the general role of the music in disease prevention, health promotion and the process of patient hospitalisation and recovery? What was the impact of music on the participant’s lived experiences with a rare disease / disease perception? How have the MT&MBI been used: as a research method or as a tool?
**Advantages**	What were the main advantages of the study?
**Limitations**	What were the main limitations of the study?
**Recommendations**	What were the recommendations and practical implications of the study?

***V. Collating, summarising and reporting the results***

The review will be conducted and reported following the guidelines suggested by JBI Reviewer’s Manual for Scoping Reviews ( Peters *et al*., 2020) and PRISMA Extension for Scoping Reviews ( Tricco *et al*., 2018). We will identify the relevant information according to our study aim and question, extract and chart the data, make a narrative, and summarise the findings. The PRISMA flow chart offered by Moher *et al*. (2009) will be used to illustrate the search strategy.

We will thematically analyse the studies’ findings, using qualitative descriptive methods to overview the literature as Levac *et al*. (2010) advised. Findings will be structured into thematic categories, and the key findings will be presented in sections such as the introduction; aim of the study and methodology; and results of the overviewed papers, including the following information:

- general characteristics of the studies;
- aim / -s and goal / -s;
- application of the MT&MBI;
- applied methods and data analysis;
- main results – the impact of MT&MBI on the participants' health and well-being, as well as on their lived experiences with a rare disease;
- gaps in the literature and recommendations for future research and implications for the practice.

We will provide the readers with the conclusions and identify the strengths and limitations of our research. Our study will emphasise the importance of MT&MBI for children living with rare diseases in healthcare settings. It will also inform our SAMPI research project ^1^, helping to foster new ideas in future research and practice.

***VI. Consultation***

This study is a part of the SAMPI research project, which Children’s Health Ireland implements at Temple Street Children’s University Hospital (RPAC17-05). The majority of team members in this study have experience working with children living with rare diseases, complex and chronic conditions. In this study, a cross-disciplinary consultation, as a suggested element by Levac *et al*. (2010), plays a significant role – all parties will be invited, and those who expressed interest will be involved from the beginning – in the planning of this study, selection of keywords, identification of inclusion and exclusion criteria, selection of studies, and commenting on this study protocol. All will be invited to discuss preliminary findings, validate findings and inform ideas about future research. The SAMPI research project will include the development and implementation of the music-based intervention for children living with a rare disease in Children’s Health Ireland at Temple Street Children’s University Hospital. This will consist of consultations with stakeholders (collaboration and knowledge exchange with children living with rare diseases, families, and advocates).

Finally, the authors believe that the information gained through this scoping literature review will:

- Contribute to the knowledge in the field about the use of music therapy and music as an intervention / research method / tool in healthcare settings for children living with rare disease, chronic and complex conditions;
- Provide the evidence on the impact of MT&MBI on the health and well-being of children and adolescents living with rare diseases and their lived experiences;
- Support the planning of healthcare services, community / health and well-being support initiatives, arts for health and other cross-disciplinary projects with children living with rare, chronic and complex conditions;
- Help to better plan the research in the field, including the recommendations for the practical implications. The results will also be relevant for other fields where music-based interventions / music therapy is applied, such as arts and humanities and social care.

## Research ethics

Due to the nature of this study, ethical approval is not required for this scoping review. However, this study is a part of SAMPI research project where ethical approval was previously obtained (Children’s Health Ireland Research Ethics Committee, Ref no. 17.006).

## Dissemination

This study includes the academic and health care sectors. The study results will be disseminated through international conferences and peer-reviewed articles.

## Study status

Database searches have been completed. Titles and abstracts have been screened, and full text publications were assessed against the eligibility criteria. In the next steps, these selected papers will be thoroughly analysed and the results of the article screening-selection process will be presented in a PRISMA flow diagram inspired by Moher *et al*. (2009).

## Discussion

*What this study will add:*

- This will be a first and novel scoping review to understand what types of MT&MBI in the healthcare setting for children living with a rare disease research studies exist within scientific literature;
- This study will map out and identify the methodological approaches and the impact of MT&MBI on the health and well-being of children living with a rare disease in the healthcare setting;
- The review will extend the current knowledge in the field locally and internationally;
- The study will contribute to the development of music creative modality in the overall SAMPI Research Project. This review will inform future research, practice, and policy focused on the use of MT&MBI as a research tool in children and young people’s living with a rare disease and complex illness focused research.

*The preliminary strengths of the study:*

- The review will include both quantitative, qualitative and mixed methods studies, as well as case examples and will explore the evidence of the use of MT&MBI in a health care setting for children living with a rare disease and its impact with a primary focus on their health and well-being and lived experiences with a disease;
- At least two researchers (including arts for health researcher and experienced music therapist) will be involved in the design of search strategy, selecting the keywords and databases;
- At least three researchers (including arts for health researcher and experienced music therapist) will be included in designing the inclusion and exclusion criteria, selecting the studies;
- The study has the inclusion and exclusion criteria to extract the relevant studies which have an impact on making the results more reliable and strengthening the theoretical base of the study;
- The development of a studies characteristics overview framework will make the presentation of the finding more systematic.

## Data availability

No data are associated with this article.
