# Supplementary material for: The use of music for children and adolescents living with rare diseases in the healthcare setting: a scoping review study protocol
Source: HRB Open Res. 2022 Apr 29;4:52. Originally published 2021 May 14. [Version 2] doi: 10.12688/hrbopenres.13280.2 (PMC9607902; doi:10.12688/hrbopenres.13280.2)
Supplement: Peer review history

# Peer review: Reviewer response for version 2

**DOI:** 10.21956/hrbopenres.14800.r32176

**Published:** 2022-06-16

**Authors:** Fran Garry

**Affiliations:** 1 Irish World Academy of Music and Dance and Health Research Institute, University of Limerick, Limerick, Ireland

Thank you for the opportunity to review this study protocol. The paper provides a strong case for the review in terms of the paucity of research around the use of music therapy and music-based interventions to support children with rare diseases in healthcare settings. The various steps for undertaking a scoping review are outlined. I have listed some observations and suggestions aimed at enhancing clarity for the reader below:

 Methods

 Identifying the research question:

- Could the authors elaborate briefly on what is meant by ‘MT & MBI as a research method’, as distinct from ‘a tool to support children’s health and wellbeing...’ and also clarify any key differences between MT & MBI?
- The study question is: ‘What are the roles and benefits of MT & MBI as a tool for researching children living with rare disease in healthcare settings?’ However, throughout the paper, the authors refer mostly to ‘impacts’, which seems more open to the inclusion of possible challenges for researchers and participants. Could the authors clarify if the focus is on positive impacts only?

Search Strategy and Study Selection:

- Could the authors clarify if preliminary searches were carried out in a specific database or databases?
- The phrase ‘..make necessary refinements to the search strategy’ seems to belong in the previous paragraph where the search strategy is discussed, rather than in the paragraph referring primarily to screening and study selection. Will the reviewers trial the preliminary data extraction form (Table 3) on an initial number of studies?

Charting the data:

- Regarding the preliminary data extraction chart: ‘Table 3. Initial table of the overview of study findings: the characteristics of the studies’, could I kindly request that the authors include a citation in the revised version for Garry *et al. *(2020) ^1^ in relation to the specific music focused questions presented in the chart? The full reference for this published study protocol is: Garry F, Murphy Tighe S, MacFarlane A, Phelan H. The use of music as an arts-based method in migrant health research: a scoping review protocol [version 1; peer review: 2 approved]. HRB Open Research. 2020;3(75) doi:10.12688/hrbopenres.13121.1 I wish to clarify that this refers to my own work and the work of my co-authors. The music related and project focus questions comprised our original additions to the table edited from JBI Reviewer’s Manual, 11.2.7 Data extraction (Nittas *et al.* 2018 ^2^; Peters *et al.* 2017).

Collating, summarising and reporting the results:

- The authors say that the PRISMA flow chart will be used to illustrate the search strategy. I suggest changing this to match the purpose of the flow chart as outlined correctly further on in the ‘Study Status’ section.

Preliminary strengths of the study:

- The authors write in future tense about the selection of the inclusion/exclusion criteria but they have already stated that this process was completed in the ‘Identifying relevant studies’ section. Could this be clarified further?

Overall, this is clearly an important and well thought out study, and it is evident that the reviewers have the expertise to carry out the scoping review in accordance with the steps they have outlined.

Is the study design appropriate for the research question?

Yes

Is the rationale for, and objectives of, the study clearly described?

Yes

Are sufficient details of the methods provided to allow replication by others?

Partly

Are the datasets clearly presented in a useable and accessible format?

Not applicable

Reviewer Expertise:

Arts-based research and migrant health and wellbeing, community music research, arts-practice research, scoping review

I confirm that I have read this submission and believe that I have an appropriate level of expertise to confirm that it is of an acceptable scientific standard, however I have significant reservations, as outlined above.

## References

[1] : The use of music as an arts-based method in migrant health research: a scoping review protocol. *HRB Open Res* .2020;3: 10.12688/hrbopenres.13121.1 75 10.12688/hrbopenres.13121.1 33954279PMC8063534

[2] : Electronic patient-generated health data to facilitate prevention and health promotion: a scoping review protocol. *BMJ Open* .2018;8(8) : 10.1136/bmjopen-2017-021245 10.1136/bmjopen-2017-021245 PMC608930830099392

# Peer review: Reviewer response for version 1

**DOI:** 10.21956/hrbopenres.14453.r30261

**Published:** 2021-11-01

**Authors:** Karen Gardner

**Affiliations:** 1 Public Sector Research Group, University New South Wales, Canberra, ACT, Australia

The article outlines the approach to conducting a scoping review of the literature on the use of MT&MBI with children who have rare diseases in the healthcare setting, following the methods outlined by Arksey and O'Malley. Overall the approach to review is appropriate but I found a couple of areas confusing and would benefit from further details to help the reader understand how the methods will support the research to answer the research questions.

 It would be useful to provide a rationale for why the search dates back only to 2010. Is there any specific reason this date was decided upon?

 I found the discussion on key words and search strategy confusing. The key words for the search strategy seem to be presented in Table 2 but the rest of the paragraph discusses the processes that will be used to select these. Please clarify how these are related. 

 The second paragraph under the heading "V. Collating, summarising and reporting the results" seems to confuse reporting and analysing results. How will you actually analyse the data from extracted studies - you say using thematic analysis - can you elaborate?

 It isn't clear to me how will your analysis will support you to answer your research questions. For example, the list of items you will report on refers to impacts of programs on participants health and well being but your research questions are much broader than this and include characteristics of MT&MBI programs; different types of interventions; outcomes etc . Will you use a framework to guide this thematic analysis so you can categorise characteristics of MT&MBI programs; different types of interventions; outcomes?

Is the study design appropriate for the research question?

Yes

Is the rationale for, and objectives of, the study clearly described?

Yes

Are sufficient details of the methods provided to allow replication by others?

Partly

Are the datasets clearly presented in a useable and accessible format?

Not applicable

Reviewer Expertise:

Health service research, scoping review, evaluation

I confirm that I have read this submission and believe that I have an appropriate level of expertise to confirm that it is of an acceptable scientific standard, however I have significant reservations, as outlined above.

# Response

**Published:** 2021-12-07

**Authors:** Simona Karpaviciute

**Affiliations:** Children’s Health Ireland at Temple Street Children’s University Hospital, Ireland

Dear Karen Gardner,

 Thank you for reviewing our paper. Please find our comments to your questions:

1. The period limit was applied to reveal the present-day literature.
2. In table 2 we present the selected keywords and in the two paragraphs above the description of their selection process.
3. Yes, we will use the thematic analysis approach to analyse the data. We will apply the guidelines suggested by Braun & Clarke (2006).
4. In this protocol study, the list of research questions will help to reveal the diverse aspects of selected studies. We believe, it will be beneficial for other researchers in the field to better understand the importance and varied impacts of MT & MBI. Yes, we will use a framework to guide the thematic analysis.

# Peer review: Reviewer response for version 1

**DOI:** 10.21956/hrbopenres.14453.r29476

**Published:** 2021-05-25

**Authors:** Julie McMullan

**Affiliations:** 1 Queen's University Belfast, Belfast, UK

This study is very much needed and appears to be exploring an area of support and therapy which very little is known about at this time. Much thought has been given to identify the study question with various individuals from relevant backgrounds bringing their experiences and skills to this matter. It is obvious that much thought is also being given to the methodology being used and the types of key words that will be used to conduct the searches. The level of detail is appropriate and would enable replication to be conducted with ease. The authors have endeavored to be thorough in their protocol and demonstrate a wealth of experience and knowledge to enable them to undertake such a review.

Is the study design appropriate for the research question?

Yes

Is the rationale for, and objectives of, the study clearly described?

Yes

Are sufficient details of the methods provided to allow replication by others?

Yes

Are the datasets clearly presented in a useable and accessible format?

Not applicable

Reviewer Expertise:

Public health.

I confirm that I have read this submission and believe that I have an appropriate level of expertise to confirm that it is of an acceptable scientific standard.
